# Review of the Memorandica Medica; or, Note-Book of Medical Principles

**Published:** 1860-11

**Authors:** V. H. Taliaferro

**Affiliations:** Columbus, Ga.


*Review of the Memorandica Medica; or, Note-Book of Medical Principles.* By Henry Hartshorne, A. M., M. D., Prof. of Theory and Practice of Medicine in the Medical Department of Pennsylvania College, &c., &c. Philadelphia: J. B. Lippincott & Co. By V. H. Taliaferro, M. D., of Columbus, Ga.

The work before us is intended as a "*memoranda* of the most important points of the fundamental and introductory portion of the course of lectures delivered by the author in the Department of the Theory and Practice of Medicine." "It aspires only to a position *intermediate* between the Professor's skeleton synopsis or sylabus, and the elaborate treatise or text-book," and is intended principally for the use and benefit of medical students during the period of their "attendance upon lectures, for which we think it well adopted. To this little volume we may justly apply the term, "*multum* in *parvo.*"

Part 1st is a well arranged and condensed consideration of Etiology, embracing the following classifications:

"HEREDITARY: e. g. in tuberculosis, gout, epilepsy, insanity, cancer.

DYNAMIC, or functional (by excess or defect of action), e. g. over exertion, over excitement, emotion, loss of rest, indolence, sensual excess.

MECHANICAL: e. g. wounds and injuries, tight lacing, position.

OBSTRUCTIVE: e. g. uncleanliness, ill-ventilation, neglect of bowels.

CONDITIONAL: e. g. extremes of heat or cold, partial exposures or sudden vicissitudes of temperature, moisture or dryness, electrical disturbances.

INGESTIVE: e. g. poisoning, improper diet, intemperance, abuse of medicine, starvation.

CONTACTIVE: e. g. syphilis, gonorrhœa, itch, hydrophobia, small pox, &c.

ATMOSPHERIC: e. g. miasmatic powers, cholera, yellow fever, erysipelas, &c."

One or more of these causes may exist for the production of an attack of disease, making necessary, therefore, some subdivisions which may exist "rather mentally than actually."

New names, new terms, &c., for old and established ones, we think decidedly objectionable for students, as this tendency must be to confuse rather than elucidate. The term *sarcous* temperament, which our author proposes for *bilious*, we think fully as insignificant, if not more so, in its applicability. The term *hyperæmæsthesia*, proposed for *chronic inflammation*, is objectionable for students and unnecessary to the science of medicine. We cannot regard the term, "chronic inflammation," a "*misnomer*," but significant in its application, and appropriate to that particular condition in which it is used, in contradistinction to *acute inflammation.*

In discussing the etiology of fevers, cholera, &c., the following remarks are made, and commends themselves with force and interest, not only to the student of medicine, but to the profession everywhere. In our great haste to cure disease, we are too forgetful of its original great cause, and the means in our power for the prevention of malignant and wide-spread epidemics:

"All MALIGNANT epidemics are preventable, and should be prevented, by attention to those laws of public and domestic hygiene, now becoming systematised in the sanitary preventive measures and policy. In the protection of localities from yellow fever, for instance, I believe that the *only available and desirable quarantine* is a *dirt* quarantine. Annihilate or exclude *dirt, i. e.* putrifiable animal and vegetable detritus, and *no migratory or malignant* morbific poison can sustain its existence, even if its cause be imported to the spot.

It is interesting to know that this view can be shown to be correct, even as regards *plague*, which was once looked upon as the very *type* of contagion.

Plague has now almost died out in those cities of the east to which it was formerly a frequent visitant, if not an endemic. Long ago it was finally excluded from all the parts of Western Europe; *not* by quarantine, which has always failed of its purpose, but by improved sanitary arrangements.

Further than this, *Cairo* affords an illustration of the *dependence of Plague upon* LOCAL *conditions of* MALARIA. This great oriental capitol, before the time of Mahammed Ali Pasha, many times lost tens of thousands of its inhabitants by visitations of plague. That wisely viceroy, by costly drainage, transformed an immense swamp, in the heart of the city, and the receptacle of all its filth, into a park or square (the esbekieh); and now *Cairo is free from plague.*"

Next in the order of arrangement comes PART II.: SEMEIOLOGY, which is subdivided into SECTION 1.—Symptomatology. SECTION 2.—PHYSICAL DIAGNOSIS.

Semeiology is an important subject in the great field of medical science, but is secondary to that of etiology, which leads us, as a beacon light, to triumphant success in the management of disease. Symptoms are valueless only as they guide us unerringly to the true pathological condition, or develop the primary cause of the disturbance. We should, therefore, take *together all* the symptoms of a case, though one single *prominent* symptom may satisfy the mind of the real condition. The student and *young practitioner* should, therefore, *familiarize* themselves with *all* the leading landmarks to pathological conditions. For an example, "the pulse, in disease, may be *natural, or strong, weak, firm, yieldnig, full, small, bounding, compressible, rapid, slow, quick, jirking, hard, soft, tense, gascous, corded, wiry, thready, imperceptible, regular, irregular, intermittent, dicrotous.*"

Between these conditions of the pulse important differences exist and an *educated* touch is requisite to give them their proper significance as pathognomonic indications.

The BLOOD itself is perhaps the most important of all subjects of inquiry in connection with disease. Little, however, as yet, is known of its morbid changes. The principal facts are, that

In *anœmia,* there is a deficiency of the red corpuscles.
In *plethora,* an excess of the red corpuscles.
In *leucocymemia,* an excess of the colorless corpuscles.

In *inflammation*, and in *chlorosis*, excess of fibrin.

In *gout*, excess of uric acid.

In *Bright's diease*, excess of urea, etc.

In *diabetes*, excess of sugar.

In *malignant cholera*, deficiency of water, albumen and salts."

By far, too little is known of the *conditions* of the blood in disease. The morbic changes which the blood undergoes in *fevers*, typhoid, &c., and in the different stages of fever, would doubtless throw a flood of light upon *obscure points*.

There are many important indications to disease, which are, to a great degree, neglected by the masses of professional men, either for the want of the knowledge, or the *means* of investigations. The urine, its general appearance —capacity or clearness—specific gravity—acidity or alkalinity—all have important insignifications.

"SEDIMENTS occur in the urine, either when first passed or after standing, from its containing substances insoluble in the fluid, or precipitated by cold, or from chemical changes rapidly occurring in it. Such sediments may be examined both chemically and microscopically; but it is only in a few instances that the employment of the microscope is indispensable."

"A *fawn-colored* deposit, *entirely dissolved by heat*, consists of URATES of ammonia and soda. A similarly colored deposit of cystine occurs rarely, *not soluble by heat*.

*Heavy red sand* at the bottom of the vessel, insoluble in *hydroclorie acid*, but *dissolved by nitric acid*, and also by *alkalines*, (as liquor potassæ,) is URIC (lithic) acid.

*Blood corpuscles* sometimes fall to the bottom, but they are *not soluble in acids or alkalines*.

A *whitish deposit, not at all dissolved by heat,* but dissolved by nitric acid, consists of EARTHY SALTS, phosphatic or oxalic. If oxalate of lime, it will not be dissolved by acetic acid ; if phosphatis, that acid will render the liquid clear.

A creamy white or yellow, floculent and ropy deposit, dissolved by liquid potassæ consists of pus."

"*Kyestein* is a greasy pellicle found on the surface of the urine after a day or two's standing, in pregnant females, or in those whose mammary glands are excited by sympathy with uterine irritation."

Our author's remarks upon the urine—its general character and appearance, chemical tests, &c.—aside from any other consideration makes his "memoranda medica" invaluable, not only to the student of medicine, but the general practitioner."

*Physical diagnosis* is considered under appropriate heads: *Inspection*, *Mensuration*, *Palpation*, *Succussion*, *Spirometry*, *Percussion*, *Auscultation.*

PART 3.—PUERPERAL PATHOLOGY is now considered, commencing with the following division of the SEAT of disease:

"In the *constitution:* e. g. secondary syphilis, tuberculosis.

In special *tissues:* e. g. mollities osseum.

In particular *apparatus:* e. g. dyspepsia, hysteria.

In individual *organs:* e. g. pneumonia, chirrosis, hydatids.

In the *blood:* e. g. anæmia, scorbutis, cholera."

Under the head, Kasology, Part IV., Cullins' classification of diseases is subdivided into,

Phlegmasia—inflammations.
Zymoses—zymotic diseases.
Cachexsæ—cachectic affections.
Neuroses—nervous disorders.
Ataxiæ—unclassifiable diseases.

Part V. is devoted to GENERAL THERAPEUTICS in which an interesting and concise "memoranda" is given. Our author writes: "BLOOD-LETTING, *vènesection*, *leeching*, and cupping, is one of the oldest, and has been one of the most universal of remedies for inflammation. Although blood-frarers have occasionally appeared in all ages and nations, yet the aggrè-gate testimony of the proposition, from Hippocrates down to the present time, has been in favor of the use of the lancet and of local blood-letting in the treatment of violent inflammations and congestions."

Now, however, it must be admitted that blood-letting has more opponents and fewer defenders than at any previous period in medical history. Why is this? By reason of—

1.—Reaction from previously existing abuse of the remedy.

2.—A change in the average human constitution, occurring under the artificial habits of civilized life.

3.—False construction and misapplication of recent science.

4.—Leadership and fashion.

I must briefly remark, that the reaction alluded to, has proceeded too far—going from one extreme to another.

During the past half century the frequency and popularity of blood-letting has been gradually on the decline, until, doubtless, we have gone into an unnecessary, and perhaps, dangerous extreme; yet, we are well apprised, that the "change in the average human constitution, occurring under the artificial habits of civilized life," has established, to some degree, a necessity for this extreme.

The present mode of life in civilized and refined society, has so depraved the physical constitutions of our people, that it becomes necessary now to adopt a sustaining treatment, whereas, formerly, the indications led to active depletion (the most important being venesection) the successful, results of which established beyond controversy the legitimacy of such means.

The author concludes his memoranda with the following maxims:

1.—All pathology is but the physiology of organic perturbation.

2.—Never interfere actively in disease, without a direct object.

3.—Act only upon scientific reason, or well-defined experience.

4.—Treat the cause of disease when possible.

5.—Watch always, and treat when requisite, the condition of the patient.

6.—Avoid, especially, routine treatment, (an error, by the way, into which too many fall.—V. H. T.) according to the names of disease.

7.—Use no violence with self-limited diseases.

We will conclude by saying, that we are pleased with the work before us, and sincerely hope the "Memoranda Medica" may find, as it justly merits, a place in every physician and student's library.

