# Errata

**Published:** 1860-12

**Authors:** 


### Errata.

In vol. 3d, page 134, 18th line from top, for "*powers*" read poisons. Page 136, 2d line from top, for "*wisely*" read wise. Page 137, 9th line from top, for "*morbic*" read morbid; same page, 17th line from bottom, for "*insignifications*," read significations; same page, 4th line from bottom, for "*hydrochlorie*," read hydrochloric. Page 138, 5th line from top, for "*phosphatis*," read phosphatic; same page, 9th line from bottom, for "*chirrosis*" read cirrhosis. Page 139, 6th line from top, for "*blood-frearers*" read blood-fearers.

